# Genome Mining of Three Plant Growth-Promoting *Bacillus* Species from Maize Rhizosphere

**DOI:** 10.1007/s12010-021-03660-3

**Published:** 2021-09-16

**Authors:** Oluwaseyi Samuel Olanrewaju, Modupe Stella Ayilara, Ayansina Segun Ayangbenro, Olubukola Oluranti Babalola

**Affiliations:** Food Security and Safety Niche Area, Faculty of Natural and Agricultural Sciences, North-West University, Mmabatho, 2735 South Africa

**Keywords:** *Bacillus*, Biosynthetic gene clusters, Comparative genomics, Functional genomics, Pan-genome analysis, Plant growth-promoting bacteria

## Abstract

*Bacillus* species genomes are rich in plant growth-promoting genetic elements. *Bacillus subtilis* and *Bacillus velezensis* are important plant growth promoters; hence, to further improve their abilities, the genetic elements responsible for these traits were characterized and reported. Genetic elements reported include those of auxin, nitrogen fixation, siderophore production, iron acquisition, volatile organic compounds, and antibiotics. Furthermore, the presence of phages and antibiotic-resistant genes in the genomes are reported. Pan-genome analysis was conducted using ten *Bacillus* species. From the analysis, pan-genome of *Bacillus subtilis* and *Bacillus velezensis* are still open. Ultimately, this study brings an insight into the genetic components of the plant growth-promoting abilities of these strains and shows their potential biotechnological applications in agriculture and other relevant sectors.

**Supplementary Information:**

The online version contains supplementary material available at 10.1007/s12010-021-03660-3.

## Introduction

The challenges of climate change and urbanization impact on food production has necessitated the need to bring up solutions that will mitigate these effects. The health hazard posed by chemical fertilizers is a cause for concern; hence, the need for an environmental and health-friendly approach. This led to the use of microorganisms for food production. These microorganisms, termed plant growth-promoting bacteria (PGPB), increase food production through direct and indirect means such as phytohormone production, nitrogen fixation, cyanide production, siderophore production, antibiotic production, and phosphorous solubilization. These mechanisms have been reviewed in various studies [25, 31, 39, 43, 47]. They produce antifungal and antibacterial compounds to help plants against pathogens. Furthermore, they act in the bioremediation of contaminated soil. Contaminations in the form of salinity and heavy metal pollution are taken care of by PGPB. Several genus of bacteria support plant growth promotions; however, this study will focus on the *Bacillus* genus.

*Bacillus* genus is abundant in the rhizobiome. They are present in the rhizosphere of several crop plants where they support plant growth. They are good root colonizers and produce several metabolites, which makes them good biocontrol agents against plant pathogens. Reports on their biocontrol activities have been studied by various groups of researchers [3, 15, 21, 46, 50]. The most important feature of *Bacillus* spp. is their ability to form spores. Spores help to protect them against various stress conditions, thus, enabling their application for plant growth promotion, bioremediation, and industrial applications.

Furthermore, comparative microbial genomics based on sequence similarity will help in identifying the important genetic contents shared among all plant growth promoting isolates as well as subset of genes encoding biocontrol and novel functions. Pan-genome represents species genomic diversity and it includes both the core and variable genome content [11]. Pan-genome aids in taxonomic classifications (phylogenomic analysis), precise determination of genomic contents of a group (calculation of core, pan, and variable genome), and organism’s lifestyle (allopatric or sympatric) [13].

This study focuses on three strains of this genus, viz. *Bacillus subtilis* A1 (BSA1), *Bacillus velezensis* A3 (BVA3), and *Bacillus subtilis* A29 (BSA29). In a previous study [38], these strains promote the growth of maize plants on the field. In vitro assay showed their efficiency in inhibiting *Fusarium graminearum*. Furthermore, they were able to solubilize phosphate, produce IAA, siderophore, protease, oxidase, and ACC deaminase enzyme, among others. In another study [36], they produce several volatile organic compounds (VOCs) that are related to their biocontrol ability. In the present study, therefore, we report the genomic characterization and the genetic basis of the plant growth-promoting traits exhibited by these strains. The presence of phages in these strains was also reported in the study. We have used comparative genomics approach to unravel the plant growth promoting and biocontrol potential of the strains in this study. The result obtained can be applied for the genetic modification of these strains for various plant growth-promoting abilities.

## Materials and Methods

### Isolation, Identification, Antimicrobial Activity, and Plant Growth Promotion Assay

Isolation of bacterial strains and identification, as well as plant growth promotion assay, are reported in a previous study [38]. The isolates are *Bacillus subtilis* A1, *Bacillus velezensis* A3, and *Bacillus subtilis* A29. In brief, 31 isolates were screened for their plant growth-promoting traits, and 3 were selected for field trials [38]. They showed improvement in maize growth compared to the control when inoculated in single and consortia.

### Genome Sequencing and Annotation

The genomic DNA was extracted from overnight cultures in LB medium [12] using a ZR soil microbe DNA MiniPrep extraction kit (Zymo Research, USA), following the manufacturer’s instructions. The DNA quality and quantity were determined using a NanoDrop Lite spectrophotometer (Thermo Fisher Scientific, CA, USA). The genomes of the strains were sequenced on an Illumina HiSeq sequencer at Molecular Research (MR DNA), Shallowater, TX. The libraries were prepared using Kapa HyperPlus kits (Roche), following the manufacturer’s user guide. The initial concentration of DNA was evaluated using the Qubit double-stranded DNA (dsDNA) high-sensitivity (HS) assay kit (Life Technologies). A total of 25 ng of DNA were used to prepare the libraries. The protocol starts with enzymatic fragmentation to produce dsDNA fragments, followed by end repair and A-tailing to produce end-repaired 5′-phosphorylated 3′-deoxyribosyladenine (dA)-tailed dsDNA fragments. In the adapter ligation step, dsDNA adapters are ligated to 3′-dA-tailed molecules. The final step is library amplification, which employs high-fidelity, low-bias PCR to amplify library fragments carrying appropriate adapter sequences on both ends. Following the library preparation, the final concentrations of the libraries were measured using the Qubit dsDNA HS assay kit (Life Technologies), and the average library size was determined using the Agilent 2100 Bioanalyzer (Agilent Technologies). *Bacillus subtilis* A1, *Bacillus velezensis* strain A3, and *Bacillus subtilis* strain A29 DNA concentrations are 114.0, 84.8, and 187.0 ng/μl, respectively, while the final library DNA concentrations are 62.0, 62.0, and 58.8 ng/μl, respectively. The average library sizes of *Bacillus subtilis* A1, *Bacillus velezensis* strain A3, and *Bacillus subtilis* strain A29 are 680, 694, and 695 bp, respectively. The libraries were pooled, diluted (to 9.0 pM), and paired-end sequenced for 500 cycles using the HiSeq system (Illumina), with an average read length of 2 × 250 bp.

The raw sequences were processed to obtain high-quality reads using the Kbase [20] platform. The quality of the reads was checked using FastQC (v.1.0.4) [6]. The reads were trimmed to remove adapters and low-quality sequences using Trimmomatic (v.0.36) [16], with the default parameters. The reads were assembled by de novo assembly using SPAdes v.3.12.0 [35], with the default parameters. Gene function prediction was performed using the Rapid Annotations using Subsystems Technology (RAST v.2.0) server (http://rast.nmpdr.org) [8].

The Genbank accession numbers are SHOB00000000, SHOC00000000, and SHOD00000000, while the BioProject accession numbers are PRJNA516328, PRJNA516332, and PRJNA516331, respectively. The Sequence Read Archive (SRA) has accession numbers SRR8540661, SRR8550002, and SRR8541016, respectively.

### Genome Mining for BGCs, Antibiotic-Resistant Genes, Virulent Factors, and Phages

The genome sequences of the selected strains were determined and mining for biosynthetic gene clusters of antimicrobial compounds, including NRPs, PKs, NRPs-PKs hybrids, bacteriocins, and terpenes, was conducted with RAST system [8, 17, 40], antiSMASH 5.0 [14], and BAGEL4 [48] using the default settings. Each draft genome was assembled into a pseudomolecule using a closely related strain as a reference before applying to the pipelines. Antimicrobial resistance genes were mined using the Resistance Gene Identifier (RGI) tool of the Comprehensive Antibiotic Resistance Database (CARD)^4^ [4] using contigs file with the parameters “Perfect and strict hits only” and “High quality/coverage.” The presence of phages was checked using phaster [7] with default settings.

### Pan-genome and Core-genome Analyses

Bacterial pan-genome analysis (BPGA) is a high-speed and highly efficient computational pipeline used for comparative genomic analysis and pan-genome construction [19]. Pan-genome and core-genome of 10 *Bacillus* species (Table 3) were obtained by BPGA pipeline. Power-law regression based on Heaps’ law was used to calculate curve fitting of the pan-genome as follows:

*y* ¼ A pan *x*^ðB panÞ þ C pan.

where *y* is the pan-genome size, *x* is the genome number, and A_pan, B_pan, and C_pan are fit parameters. B_pan is equivalent to the parameter *c* and *a* = 1 − *c*. According to Heaps’ law, a pan-genome is open when 0\*c*\1 and *a B* 1 and close when *c*\1 and *a*[1. The exponential regression model *y* ¼ A core *e*^ðB core _ *x*Þ þ C core was used to calculate curve fitting of the core-genome, where *y* is the core-genome size, *x* is the genome number, *e* is the Euler number, and A_core, B_core, and C_core are fit parameters [45].

USEARCH clustering algorithm with a 50% cut-off was utilized for clustering genes. Pan- and core-genome plot was generated using the default settings while MUSCLE was used for aligning genes and phylogeny was analyzed with the neighbor-joining method.

### COG and KEGG Functional Classification of Genes

Downstream analysis of data subsets under KEGG/COG categories deciphers the BPGA platform for further “omics” approaches. BPGA employs the ublast function of USEARCH to identify best hits with respective reference databases and classify them according to KEGG and COG categories [19].

### Phylogenetic Analysis

Phylogenetic tree was constructed based on the average nucleotide identity (ANI). The overall similarity between the whole-genome sequences was calculated using the Orthologous Average Nucleotide Identity Tool (OAT) v0.93.1 [54].

## Results and Discussion

### General Characterization of the Strains

**Fig. 1:**
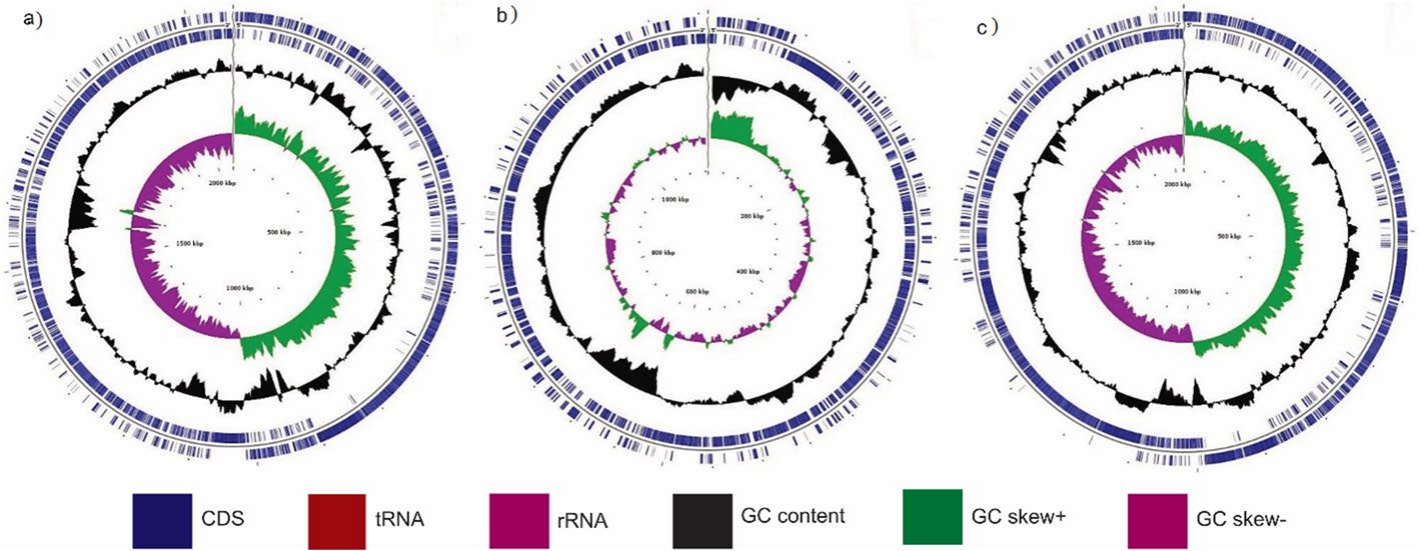
Schematic representation and general characteristics of the three *Bacillus* spp. (a) BSA1. (b) BSA29. (c) BVA3

**Fig. 2:**
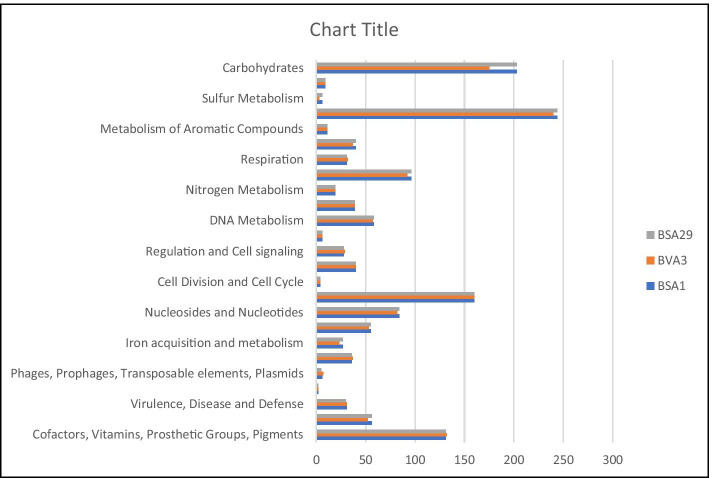
Frequency distribution of gene categories in each genome

The genome map of the three isolates presented in Fig. 1 shows the various genetic components of the isolates. Figure 2 shows the distribution of the gene categories in the isolates. In all three, gene for amino acids is more abundant followed by carbohydrate and protein metabolism.

### Genetic Elements Involved in Stress Resistance, Soil, and Plant Colonization Abilities

#### Stress Resistance and Tolerance

Genome analysis of BSA1, BVA3, and BSA29 showed the presence of stress tolerance proteins in the genomes of these isolates. Osmotic stress resistance genes of the “Opu” family are present in the three genomes (Table S1). Enzymes such as betaine aldehyde dehydrogenase and alcohol dehydrogenase that help against osmotic stress are found in the genomes. One nitrosative stress resistance gene *nsrR* is also present in all the genomes. Superoxide dismutase enzyme, which is important in militating against oxidative stress, is present in the three genomes along with *perR* and *fuR* genes. Apart from the specific stress response proteins that have been mentioned, several general stress response proteins are also present in the genomes of these microbes. These include *rsbS*, *rsbT*, *rsbW*, *rsbU*, *rsbR*, *rsbV*, and *hfq* genes. The presence of stress response genes confirms the ability of BSA1, BSA29, and BVA3 to help plants in stress tolerance [1, 50]. Proteins involved in heat and cold stress are important in DNA and RNA stabilization, thereby increasing transcription and translation efficiency during the stress period [30].

#### Spore Formation

Several genes and enzymes regulating spore formation and dormancy are largely represented in the genomes of the three microbes (Table S1). Among these are the *ypeB*, *ydhD*, *kapB*, *mazG*, *tasA*, *yqxM* genes etc. while enzymes present include sporulation kinase B and sporulation kinase C. Several spore formation gene clusters are represented in these genomes. *Bacillus* species are known for spore formation. Spore production enhances their ability to cope with environmental stress such as drought and salinity [56].

#### Heavy Metal Resistance

The genomes show the presence of copper resistance genes, viz. *copD* and *copC* genes. Proteins regulating the transportation of metals, such as the copper-translocating P-type ATPase (EC 3.6.3.4) and the cobalt-zinc-cadmium resistance protein, are present in the genomes (Table S1). The presence of heavy metal-resistant genes shows that these strains can adapt favorably when exposed to heavy metals. Heavy metals deplete bacterial populations in the soil [27], hence reducing microbial-plant growth promotion efficacy. Therefore, microbial resistance to heavy metals is critical for their survival in the soil. These strains, having several heavy metal resistance genes, stand a chance to survive in heavy metal polluted environment.

#### Motility, Chemotaxis, and Attachment to Plant Surfaces

All forms of movement in *Bacillus* spp. are with the use of flagella. The flagellum is their medium of transport for food and attachment to host plants. Therefore, it is an important feature in these bacteria genera. Based on this, the genomes of these isolates revealed the presence of several flagella biosynthesis proteins and regulators such as *flgB*, *flgD*, and *flgK*. Cell division protein Fst1, which codes for the enzyme peptidoglycan synthetase (EC 2.4.1.129), is also present in the genomes (Table S1). The ability to move and attach to plant surfaces is important for efficient root colonization. Teichoic acid is fundamental in root colonization [53]. The genomes of these strains revealed the presence of genes involved in the production of teichoic acid.

### Genetic Elements Involved in Plant Growth Promotion Activities

#### Biocontrol Activities and Antibiotic Resistance

**Fig. 3:**
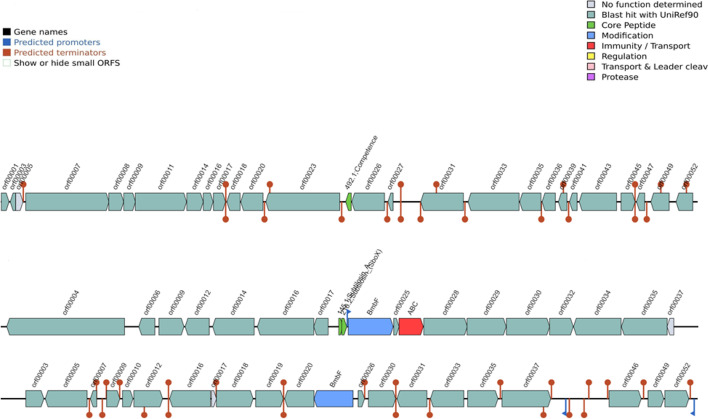
Bacteriocins detection from BAGEL 4

**Fig. 4:**
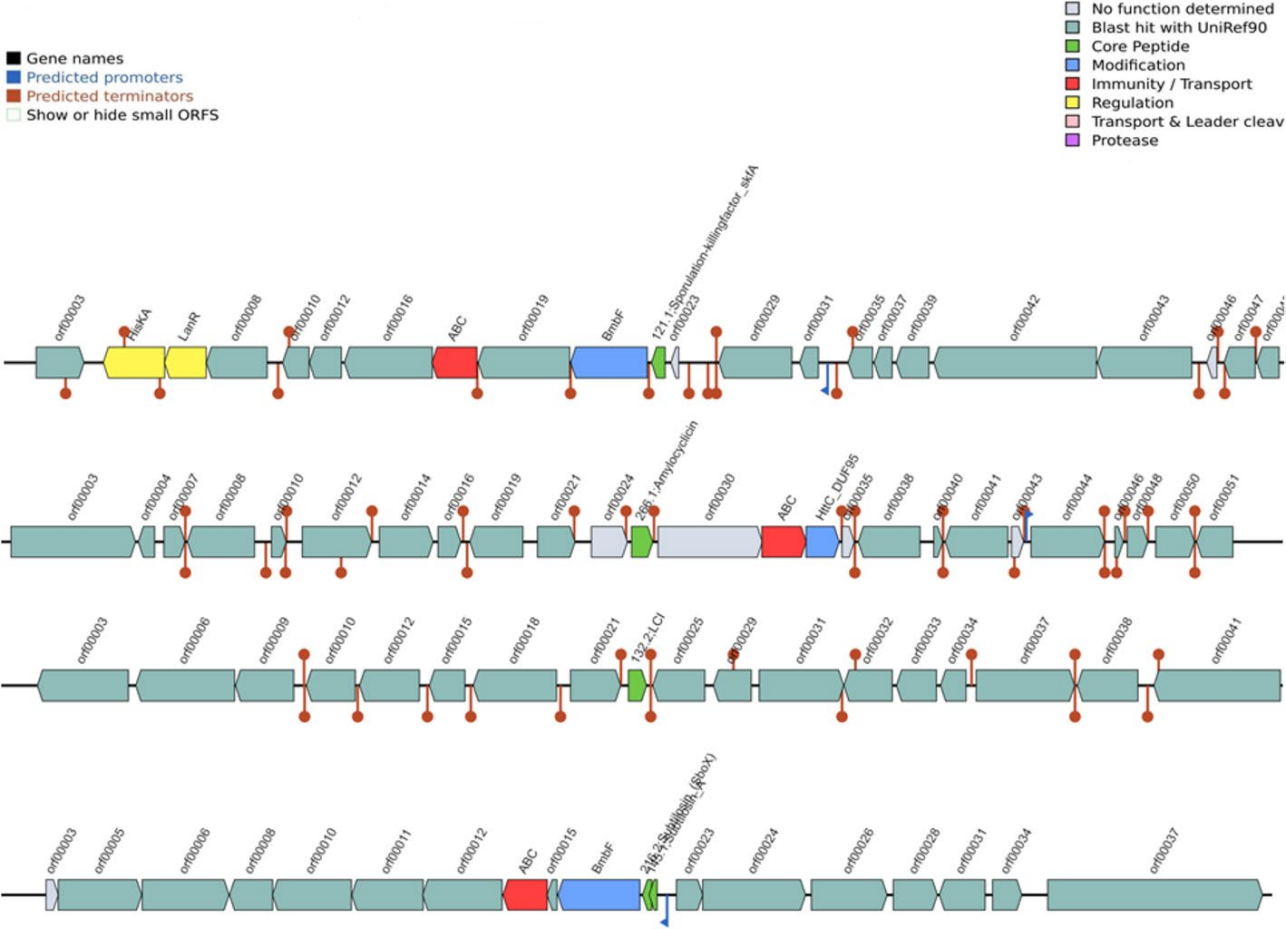
Presence of antimicrobial resistance genes in the genomes of the three isolates

Genomic analysis revealed the presence of several gene clusters involved in the production of antimicrobial compounds, including genes involved in bacteriocins, terpenes, PKS, and NRPs gene clusters (Tables 1 and S2, Figs. 3 and 4). Gene clusters involved in bacillibactin, fengycin, macrolactin H, subtilosin A, sporulation killing factor, and surfactin were reported (Tables 1 and S2, Figs. 3, 4, S1–S9). In addition, antibiotic resistance genes were detected in the genomes (Table 2, Figs. S10-S13). Genes coding for daptomycin resistance and tunicamycin resistance proteins, including the *ykkC*, *ykkd*, *aadK*, *vmlR*, and *tmrB* genes (Table 2, Figs. S10-S13); genes encoding for streptothricin resistance and Fosfomycin resistance protein (*fosB*); and genes coding for resistance to fluoroquinolones are present (Table S1). *Bacillus* strains, especially *Bacillus subtilis*, are good biocontrol agents [15, 21, 22].

**Table 1:** List of putative secondary metabolite producing biosynthetic clusters as predicted by antiSMASH

Clusters	Regions	Size (bp)	Most similar known biosynthetic cluster	Similarity (%)	MIBiG BGC-ID
BSA1					
Hybrids	1.1 (NRPS-betalactone)	83,415	Fengycin	100	BGC0001095
	3.2 (transAT-PKS-T3PKS-NRPS)	107,129	Bacillaene	100	BGC0001089
Terpenes	1.2	21,898	-	-	-
	3.1	20,806	-	-	-
PKS	1.3	41,097	-	-	-
NRPS	2.3	49,741	Bacillibactin	100	BGC0000309
	5.1	26,163	Surfactin	43	BGC0000433
	6.1	28,220	Surfactin	43	BGC0000433
	10.1	9,421	Surfactin	8	BGC0000433
Others	2.1	41,418	Bacilysin	100	BGC0001184
Sactipeptides	2.2	21,611	Subtilosin A	100	BGC0000602
	6.2	22,953	Sporulation killing factor	100	BGC0000601
BVA3					
Hybrids	1.4(NRPS-betalactone-transATPKS-bacteriocin)	136,315	Fengycin	100	BGC0001095
	1.5(transATPKS-NRPS-T3PKS)	100,576	Bacillaene	100	BGC0001089
	2.2(bacteriocin-NRPS)	51,790	Bacillibactin	100	BGC0000309
Terpenes	1.3	20,126	-	-	-
	1.7	20,740	-	-	-
PKS	1.2(T3PKS)	40,687	-	-	-
	1.1(transAT-PKS)	92,393	Difficidin	100	BGC0000176
	1.6(transAT-PKS)	88,212	Macrolactin H	100	BGC0000181
NRPS	3.1	65,407	Surfactin	91	BGC0000433
Others	2.1	41,418	Bacilysin	100	BGC0001184
BSA29					
Hybrids	1.2(transATPKS-T3PKS-NRPS)	85,277	Bacillaene	100	BGC0001089
	1.3(NRPS-betalactone)	83,415	Fengycin	100	BGC0001095
Terpenes	1.1	20,398	-	-	-
	1.4	21,898	-	-	-
PKS	1.5(T3PKS)	41,097	-	-	-
NRPS	2.1	49,741	Bacillibactin	100	BGC0000309
	4.1	26,163	Surfactin	43	BGC0000433
	5.1	28,220	Surfactin	43	BGC0000433
	9.1	9,421	Surfactin	8	BGC0000433
Others	2.3	41,418	Bacilysin	100	BGC0001184
Sactipeptides	2.2	21,611	Subtilosin A	100	BGC0000602
	5.2	22,953	Sporulation killing factor	100	BGC0000601

**Table 2:** Presence of antibiotic resistance genes

RGI criteria	ARO term	SNP	Detection criteria	AMR gene family	Drug class	Resistance mechanism	%IdMR	%LoRS
Strict	ykkC		Protein homolog model	Small multidrug resistance (SMR) antibiotic efflux pump	Aminoglycoside antibiotic, tetracycline antibiotic, phenicol antibiotic	Antibiotic efflux	98.21	100
Strict	ykkD		Protein homolog model	Small multidrug resistance (SMR) antibiotic efflux pump	Aminoglycoside antibiotic, tetracycline antibiotic, phenicol antibiotic	Antibiotic efflux	98.1	100
Strict	aadK		Protein homolog model	ANT(6)	Aminoglycoside antibiotic	Antibiotic inactivation	98.59	100
Strict	vmlR		Protein homolog model	ABC-F ATP-binding cassette ribosomal protection protein	Macrolide antibiotic, lincosamide antibiotic, streptogramin antibiotic, tetracycline antibiotic, oxazolidinone antibiotic, phenicol antibiotic, pleuromutilin antibiotic	Antibiotic target protection	98.91	100.18
Strict	tmrB		Protein homolog model	Tunicamycin resistance protein	Nucleoside antibiotic	Reduced permeability to antibiotic	97.97	100
Strict	mphK		Protein homolog model	Macrolide phosphotransferase (MPH)	Macrolide antibiotic	Antibiotic inactivation	97.06	100
Strict	*Bacillus subtilis* pgsA with mutation conferring resistance to daptomycin	A64V	Protein variant model	Daptomycin-resistant pgsA	Peptide antibiotic	Antibiotic target alteration	100	100

*%IdMR* percentage Identity of Matching Region, *%LoRS* percentage Length of Reference Sequence

The ability to produce toxic compounds against pathogens is important for the biological control of plant pathogens. *Bacillus* produce compounds such as fengycin, surfactin, bacilysin, bacillomycin, and subtilosin [2, 24]. Fengycin acts by inhibiting the growth of filamentous fungi [33]. The surfactin lipopeptides have antiviral activity and show some antifungal activity. Fengycin, bacillomycin, and surfactin act stronger in cooperation as biocontrol agents and in biofilm formation. This was concluded in the study of Zeriouh et al. [55] where they reported reduced production of biofilm due to the absence of surfactin. So much importance is attached to the antimicrobial production by *Bacillus* species in biocontrol. *Bacillus* mutants that were unable to produce surfactin, bacillomycin, and fengycin lost their ability to control various plant diseases [9].

Antimicrobial resistance is becoming a challenge to clinical, industrial, and agricultural sectors [26]. The presence of biofilm in *Bacillus subtilis*, especially, makes it a classic example in this challenge. Biofilms protect the microbes from being destroyed by antibiotics. To the plants, this helps in protecting the plant’s “fighters” from being destroyed by toxin-producing pathogens. The results in this study support and confirm that *Bacillus subtilis* [18] and *Bacillus velezensis* [32, 46] produce antifungal and antibacterial compounds for improved plant health.

#### Iron Acquisition

The three genomes contain genes involved in iron metabolism as well as siderophore biosynthesis, especially bacillibactin and anthrachelin class siderophores. Iron acquisition gene *yycJ/Wa1J* and heme transporter protein *htsABC* (Table S1) are present in the genomes of the species. Iron transport peroxidase *efeB* and permease *efeU* are present in the three genomes. Additionally, gene clusters involved in iron cluster assembly were found, and it contains *paaD*-like protein (DUF59), *iscR*, *sufE2*, *sufD*, *sufC*, *sufB*, and *apbC* genes (Table S1). Iron is important in the synthesis of chlorophyll, and it is important in the maintenance of chloroplast structure. Therefore, the availability of iron is crucial for plant’s survival. Siderophores are molecules produced by bacteria, which makes iron available for plant use [25]. Siderophore-producing bacteria improve iron availability for plant development [37]. The presence of siderophore-producing genes in the genomes of these strains shows that they will be able to sequester available iron in the rhizosphere for plant use.

#### Nitrogen, Sulfur, and Phosphorus Acquisition

Genes regulating the metabolism of nitrogen, sulfur, and phosphorous are present in the genomes of the three bacteria. Nitrite reductase, glutamine synthetase, and glutamate synthase genes are all present (Table S1). Nitrogen regulatory protein P-II class and ammonia transport proteins are present. Nitric oxide reductase genes *norD* and *norQ* are present. This shows the ability of these bacteria to counter nitrosative stress. Additionally, genes involved in sulfate metabolism were detected. Genes for disulfide reductase (Tpx and Bcp types) in all three genomes and genes for galactosylceramide and sulfatide metabolism (arylsulfatase gene) were found in the genomes of BSA1 and BSA29. Furthermore, phosphate metabolism was supported by the presence of manganese-dependent inorganic pyrophosphatase, alkaline phosphatase genes (*phoP*, *phoH*, and *phoR*), and pyrophosphatse gene *ppax* (Table S1). The major sources of nitrogen for plant are chemical fertilizers and biological nitrogen fixation [5] and nitrogen is the most limiting element in plant growth, hence the importance of the nitrogen-fixing ability in these strains. Coupled with the presence of sulfur and phosphate synthesis genes, these strains are capable of nitrogen-fixation and phosphate solubilization [23, 38]. The availability of these nutrients is essential and critical in plant growth promotion.

#### IAA Biosynthesis and Ɣ-Aminobutyric Acid (GABA) Metabolism

The three genomes reveal the presence of genes regulating auxin biosynthesis. These are tryptophan synthase, anthranilate phosphoribosyltransferase, and phosphoribosylanthranilate isomerase (Table S1). The genomes also possess *gabR* gene encoding GABA aminotransferase (Table S1). Phytohormones regulate plant growth and tolerance. Auxin, a major phytohormone, is essential for growth regulation and stress adaptation responses. GABA, on the other hand, is involved in signaling between rhizosphere microorganisms and plants [37]. The presence of IAA and GABA genes in the genomes of these isolates infers that they will be effective in mitigating plant stress while promoting plant growth and development.

#### Polyamine Production and Modulation of Ethylene Levels

Several polyamine biosynthesis and transport genes are found in the genomes of the *Bacillus* spp, including *arcD* gene encoding for arginine/ornithine antiporter, and genes encoding for enzymes involved in spermidine biosynthesis, which include spermidine synthase. Agmatinase involved in putrescine biosynthesis is also present in the genomes (Table S1). Polyamines are important in the plat growth promoting abilities of *Bacillus* strains. Xie et al. [51] reported in their study that spermidine production by *Bacillus subtilis* was found to inhibit the production of ethylene, which affects interactions between plants and microorganisms.

#### Plant Growth Promotion Activities by Modulation of VOCs

Biosynthesis of VOCs originates from sulfur, nitrogen, ketones, alcohols, and aldehyde compounds. The genome analysis of these species shows a high level of genes and enzymes involved in the biosynthesis of these compounds. Therefore, these isolates can produce a high number of VOCs. Besides, genes involved in the biosynthesis of acetoin and butanediol are present (Table S1). VOCs impact plant growth by acting as signal molecules [37] and as biocontrol agents. *Bacillus* spp. are known to produce diverse kinds of VOCs, which result from the compounds stated above. Genes and enzymes involved in the pathways of these compounds are present in the genomes of BSA1, BVA3, and BSA29 (Table S1). Genes and pathways involved in the metabolism of acetoin and butanediol are revealed by the genomic analysis. *Bacillus subtilis* and *Bacillus velezensis* are reported to be good producers of acetoin and butanediol, which are used in biocontrol activities [34, 41, 52].

### Phage Synthesis

**Fig. 5:**
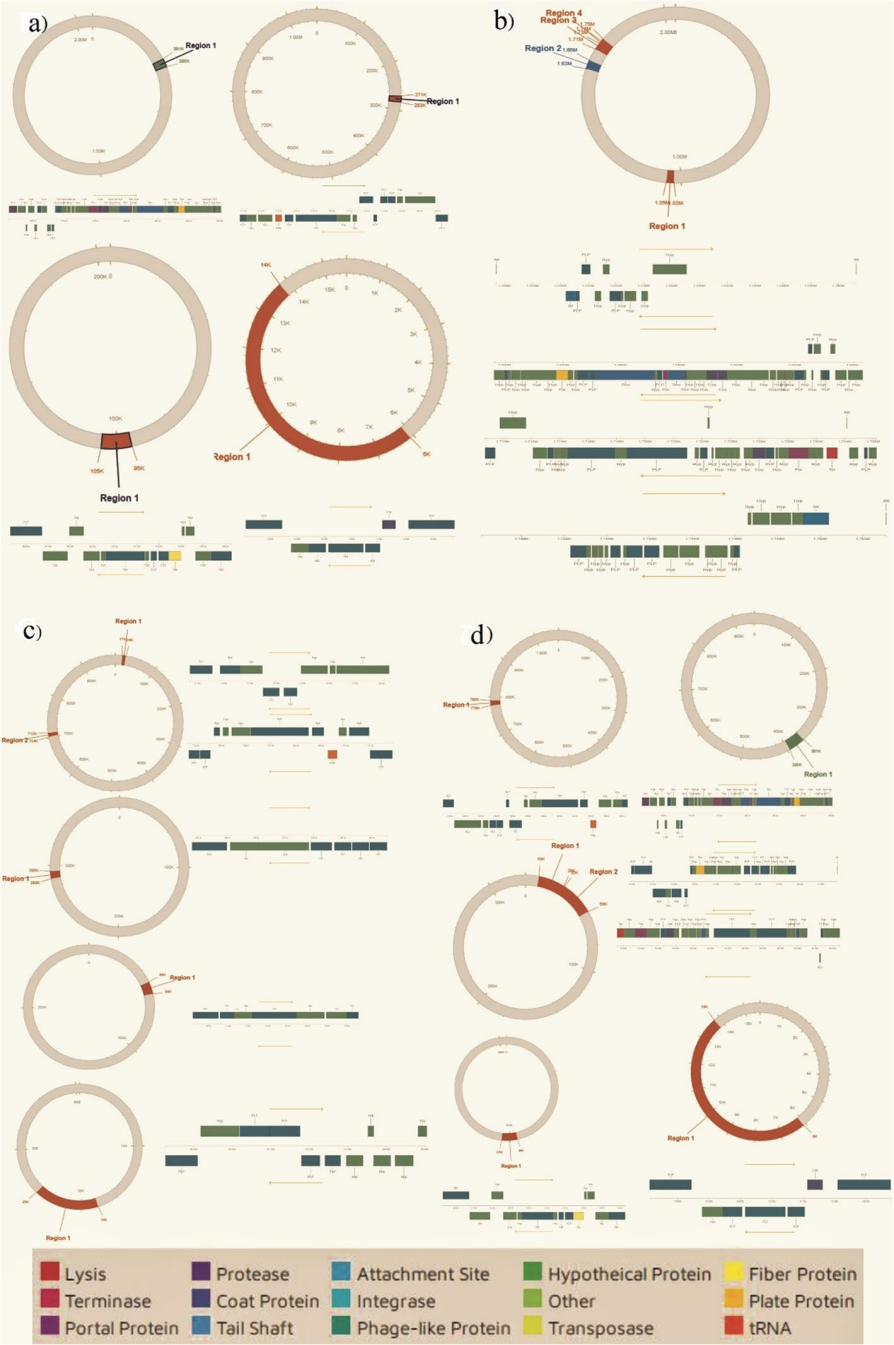
Remnants of bacteriophage regions. The boxes are color-coded with the legend pasted below the figure to show their potential functions

The genomes of the *Bacillus* spp. were investigated for the presence of prophages, plasmids, and insertion elements. The results showed at least one intact phage region for all strains (Fig. 5). Blast hits against the virus, and bacterial databases are shown in Table S3. No plasmid was found in any of the strains. Different regions and the number of coding sequences are shown in Table S3. These strains can be effectively engineered in developing phage therapies for pathogen biocontrol. Phages have been reported as good biocontrol agents against human, animal, plant, and foodborne pathogens [28, 42, 49].

### Pan-genome Analysis

Bacterial genomes harbor core and accessory genes, which may be specific to an individual species. Core-genomes are present in all strains studied while the accessory genomes differentiate one specie from another [45]. The accessory genomes usually confer genes regulating species-specific advantageous traits such as metabolite production, antibiotic resistance, virulence mechanisms, plant growth and disease suppression, siderophore production, and/or growth hormone production.

**Fig. 6:**
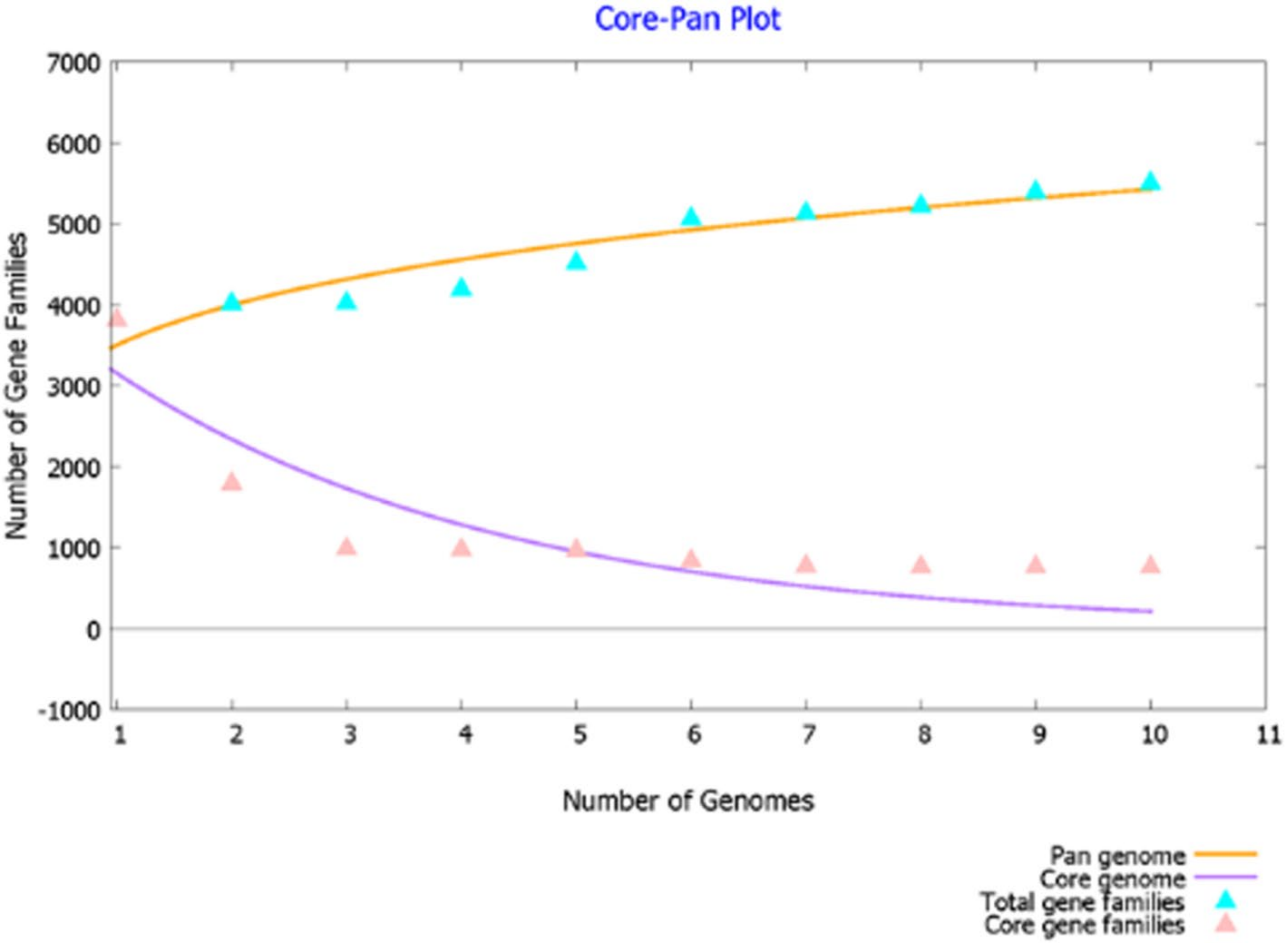
Pan- and core-genomes based on the number of sequenced genomes

In this study, we selected 10 *Bacillus* species (5 *B. subtilis* and 5 *B. velezensis*) based on their host (soil or plant) to estimate the pan- and core-genome sizes (Table S4). From our analysis, we conclude that the *B. subtilis* and *B. velezensis* have an open genome since the core/pan-genome ratio did not reach a distinct plateau (Fig. 6). However, the addition of more genomes might add to the number of accessory and unique genes, which is in line with the previous hypothesis by [10]. The pan-genome consisted of 777 core genes while BSA29 has the lowest number of accessory genes and unique genes, and *Bacillus subtilis* R31 has the highest number of accessory genes and unique genes (Table 3).

**Table 3:** Comparative genomic statistics of the species used in this study

Genome no	Organism name	Filename	Number of proteins	Genomic GC (%)	Core genes	Accessory genes	Unique genes	Absent genes
1	*Bacillus_subtilis*	*Bacillus subtilis* Bs-115	4039	43.3	777	2928	124	13
2	*Bacillus_subtilis*	*Bacillus subtilis* strain A1	2062	43.2	777	1227	13	0
3	*Bacillus_subtilis*	*Bacillus subtilis* strain A29	1157	42.8	777	361	4	657
4	*Bacillus_subtilis*	*Bacillus subtilis* strain DKU_NT_03	4082	43.2	777	2976	86	6
5	*Bacillus_subtilis*	*Bacillus subtilis* strain R31	4143	43.3	777	2992	207	1
6	*Bacillus_velezensis*	*Bacillus velezensis* strain 9D-6	3763	46.3	777	2766	94	2
7	*Bacillus_velezensis*	*Bacillus velezensis* strain A3	1899	46.2	777	1060	22	57
8	*Bacillus_velezensis*	*Bacillus velezensis* strain TB1501	3693	46.4	777	2741	61	10
9	*Bacillus_velezensis*	*Bacillus velezensis* strain WRN014	3846	46.1	777	2783	166	2
10	*Bacillus_velezensis*	*Bacillus velezensis* strain YB-130	3748	46.3	777	2750	110	4

### Phylogenomics

**Fig. 7:**
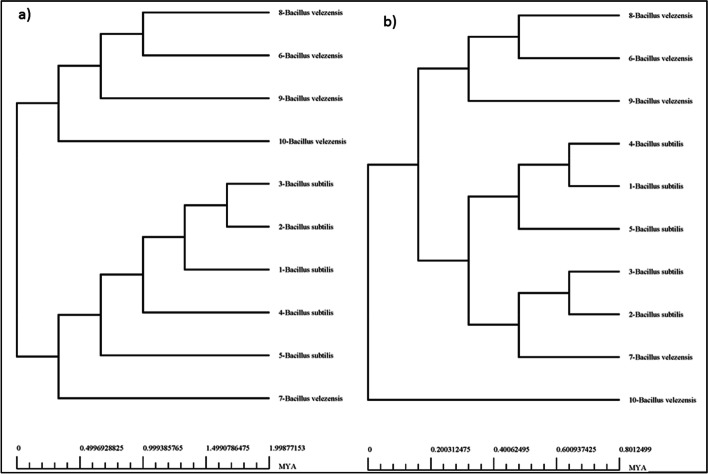
Phylogenetics of the genomes based on the (a) core- and (b) pan-genomes

**Fig. 8:**
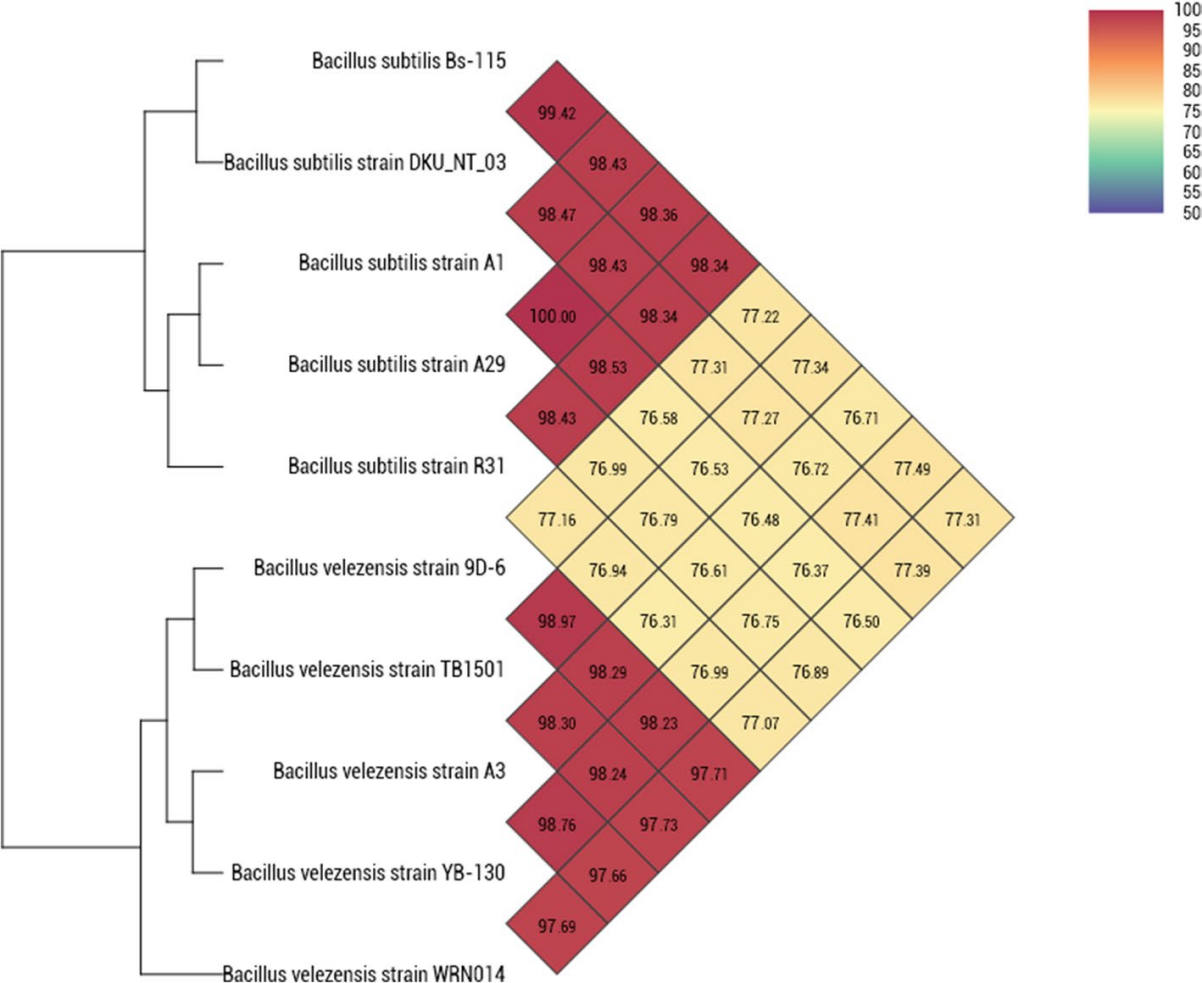
Average Nucleotide Identity (ANI) demonstrating nucleotide-level genomic similarity between the coding regions of indicated *Bacillus subtilis* and *Bacillus velezensis* genomes. Pairwise comparisons for all 10 genomes were computed using the OAT Program

Our phylogenetic trees were based on analyses of the core-genome, and ANI of the 10 genomes used (Figs. 7 and 8). The findings further refine the relationships within the genus. The phylogenetic tree based on the core-genomes (Fig. 7a) revealed that BVA3 is closer to *B. subtilis* R31 than the other *B. velezensis* species while BSA1 and BSA29 are very much close to each other same as the pan phylogeny (Fig. 7b). However, the BVA3 position in the pan phylogeny is closer to BSA1 than the other *B. velezensis* species. Therefore, we can infer that BVA3 have the same ancestor with BSA1 and BSA29.

Despite the drawback that the resolution is normally not sufficient to fully separate sub-species and the fact that that it is also liable to biases due to primer sequence matching [44], 16S rRNA sequencing is the most used parameter to explore bacteria phylogenetic relationships. Phenotypical and biochemical traits have also been used; however, these traits can be affected by choice of culture medium and other conditions. Therefore, there is need for objective methods that show high resolution. One promising method is the use of average nucleotide identity which was described by Han et al. [29]. In this study, the OrthoANI analysis showed 100% average nucleotide identity between our isolates BSA1 and BSA29 while BVA3 shows 98.76% similarity with *B. velezensis* YB-130. When comparing all isolates from this study, values were between 98.34 and 100% (for *B. subtilis* species) and between 97.66 and 98.97% for the *B. velezensis* strains. The comparison between the *B. subtilis* strains and *B. velezensis* strains showed always identity values below 80%.

### Functional Genome Analyses

To assign biological functions to the orthologs, the corresponding amino acids were annotated using COG. According to the COG analysis major category distribution, majority of the core proteins are for metabolism (40.3%), followed by information storage and processing (25.6%), 23.03% were poorly characterized while the remaining 11.01% are for cellular signaling and processing (Fig. S14a). In detail, the gene functions are as follows: cell cycle control, cell division, chromosome partitioning (2.43%), cell wall/membrane/envelope biogenesis (13.21%), cell motility(5.66%), post-translational modification, protein turnover, and chaperones (9.31%), signal transduction mechanisms (15.15%), intracellular trafficking, secretion, and vesicular transport (3.75%), defense mechanisms (10.9%), translation, ribosomal structure and biogenesis (10%), and transcription (30.64%). Others include those involved in replication, recombination and repair (21%), energy production and conversion (11.97%), carbohydrate transport and metabolism (19.02%), amino acid transport and metabolism (25.02%), nucleotide transport and metabolism (4.48%), coenzyme transport and metabolism (10.73%), lipid transport and metabolism (10.94%), secondary metabolites biosynthesis, catabolism (12.48%), inorganic ion transport and metabolism (13.74%), general function prediction only (39.04%), and genes with unknown functions (30.46%) (Fig. 10b). We also use KEGG to map cellular functions and the genes were divided according to the biological pathways they are likely to participate in (Fig. S15).

## Conclusion

*Bacillus* species have great potentials in agriculture and biotechnology. They are good producers of biocontrol agents and plant growth-promoting molecules hence their various applications for plant growth promotion and biocontrol abilities on various crops. The genomic analysis of *B. subtilis* A1, *B. velezensis* A3, and *B. subtilis* A29 showed the presence of genes, enzymes, and pathways involved in many plant growth-promoting activities such as growth hormone production, VOCs production, siderophore production, nitrogen, phosphorous, and sulfur metabolism. Their capability to be used in developing biocontrol phages was also established in the presence of many phage regions in their genomes. The pan-genome of *Bacillus subtilis* and *Bacillus velezensis* are still open. Hence, these isolates are promising plant growth promoters and can improve food security.

## Supplementary Information

Below is the link to the electronic supplementary material.

Supplementary file1 (DOCX 15.3 MB)

Supplementary file2 (XLSX 216 KB)

Supplementary file3 (XLSX 114 KB)

Supplementary file4 (XLSX 37 KB)

Supplementary file5 (XLSX 11 KB)
