# Supplementary material for: Genome Mining of Three Plant Growth-Promoting Bacillus Species from Maize Rhizosphere
Source: Appl Biochem Biotechnol. 2021 Sep 16;193(12):3949–69. doi: 10.1007/s12010-021-03660-3 (PMC8610958; doi:10.1007/s12010-021-03660-3)
Supplement: Supplementary file 1 — Supplementary file1 (DOCX 15.3 MB) [file 12010_2021_3660_MOESM1_ESM.docx]

**Genome mining of three plant-growth promoting *Bacillus* species from maize rhizosphere**

Oluwaseyi Samuel Olanrewaju, Modupe Stella Ayilara, Ayansina Segun Ayangbenro, and Olubukola Oluranti Babalola^*^

Food Security and Safety Niche Area, Faculty of Natural and Agricultural Sciences, North-West University, Mmabatho, 2735, South Africa

* Corresponding author.

E-mail address: [olubukola.babalola@nwu.ac.za](mailto:olubukola.babalola@nwu.ac.za); Telephone: +27 183892568

Supplementary information


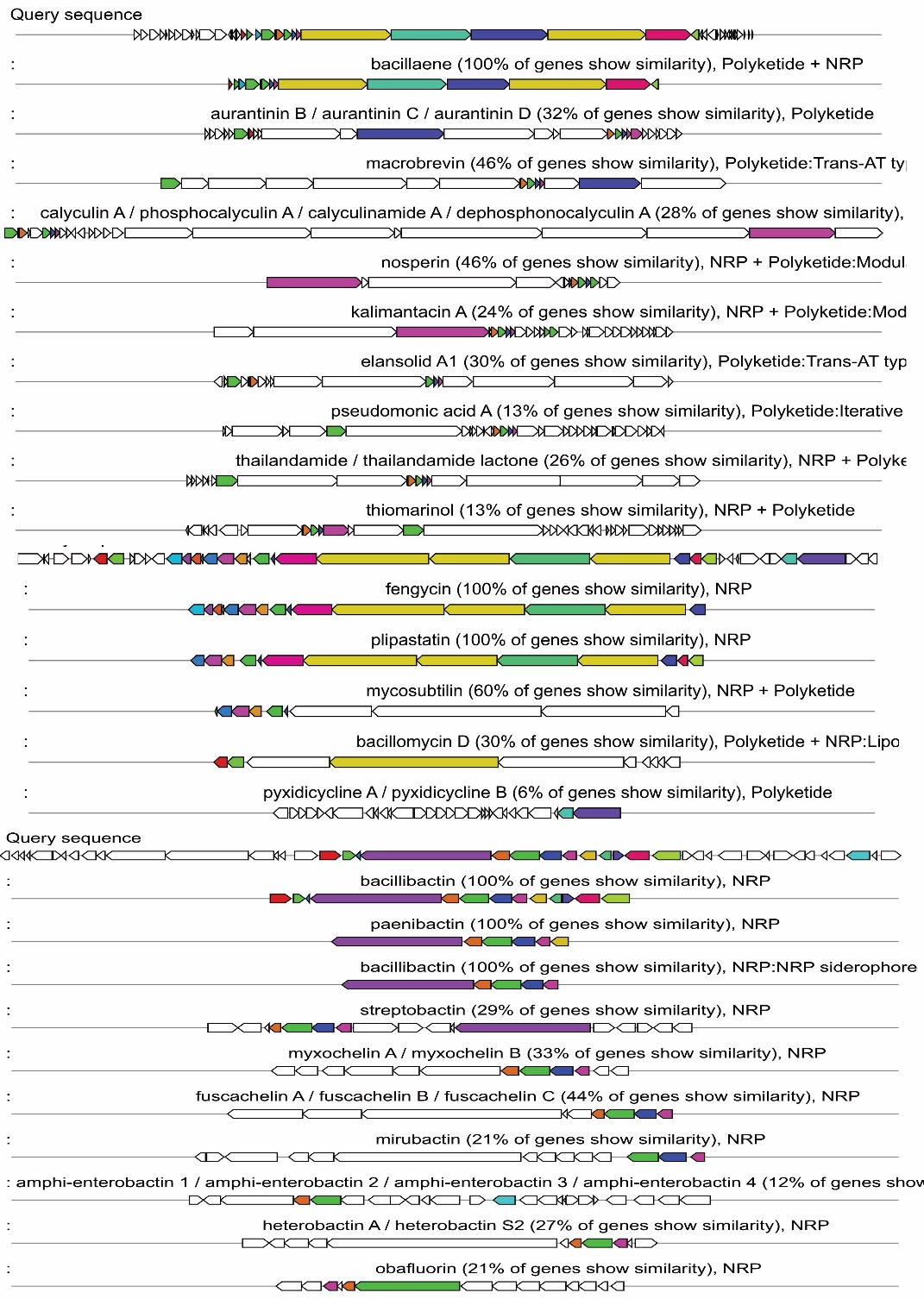


**Fig. S1** Detection of biosynthetic genes


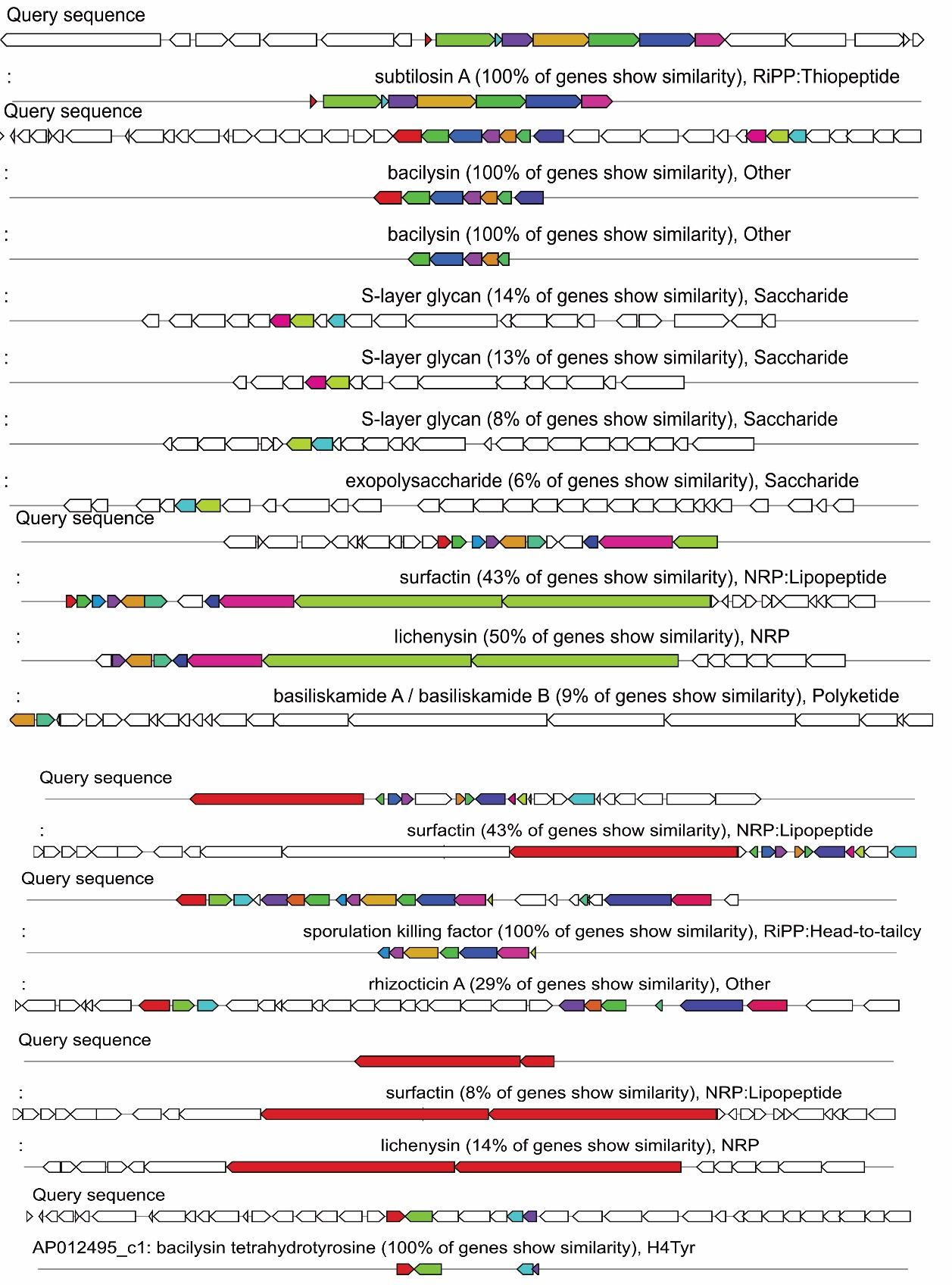


**Fig. S2** Detection of biosynthetic genes


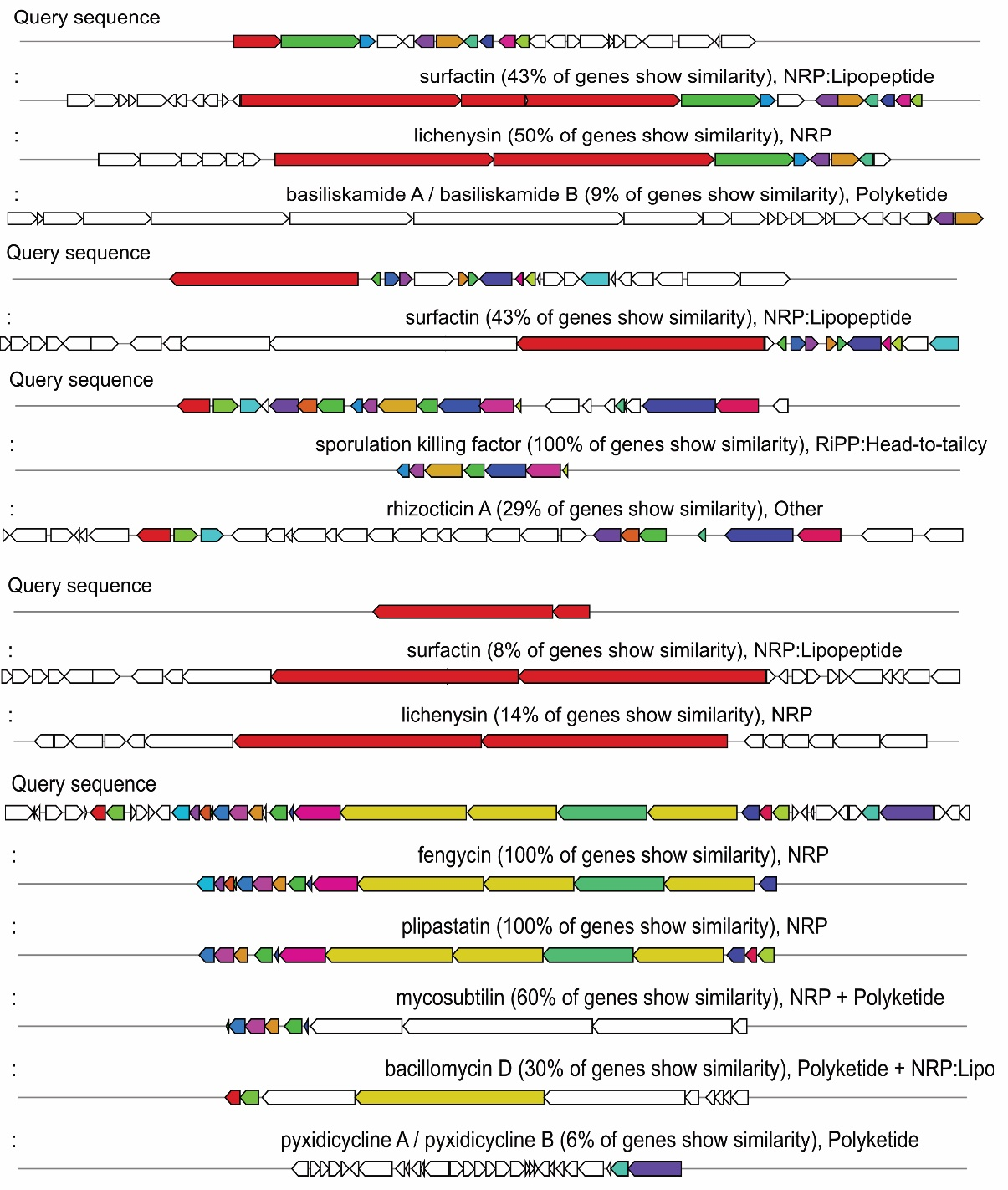


**Fig. S3** Detection of biosynthetic genes


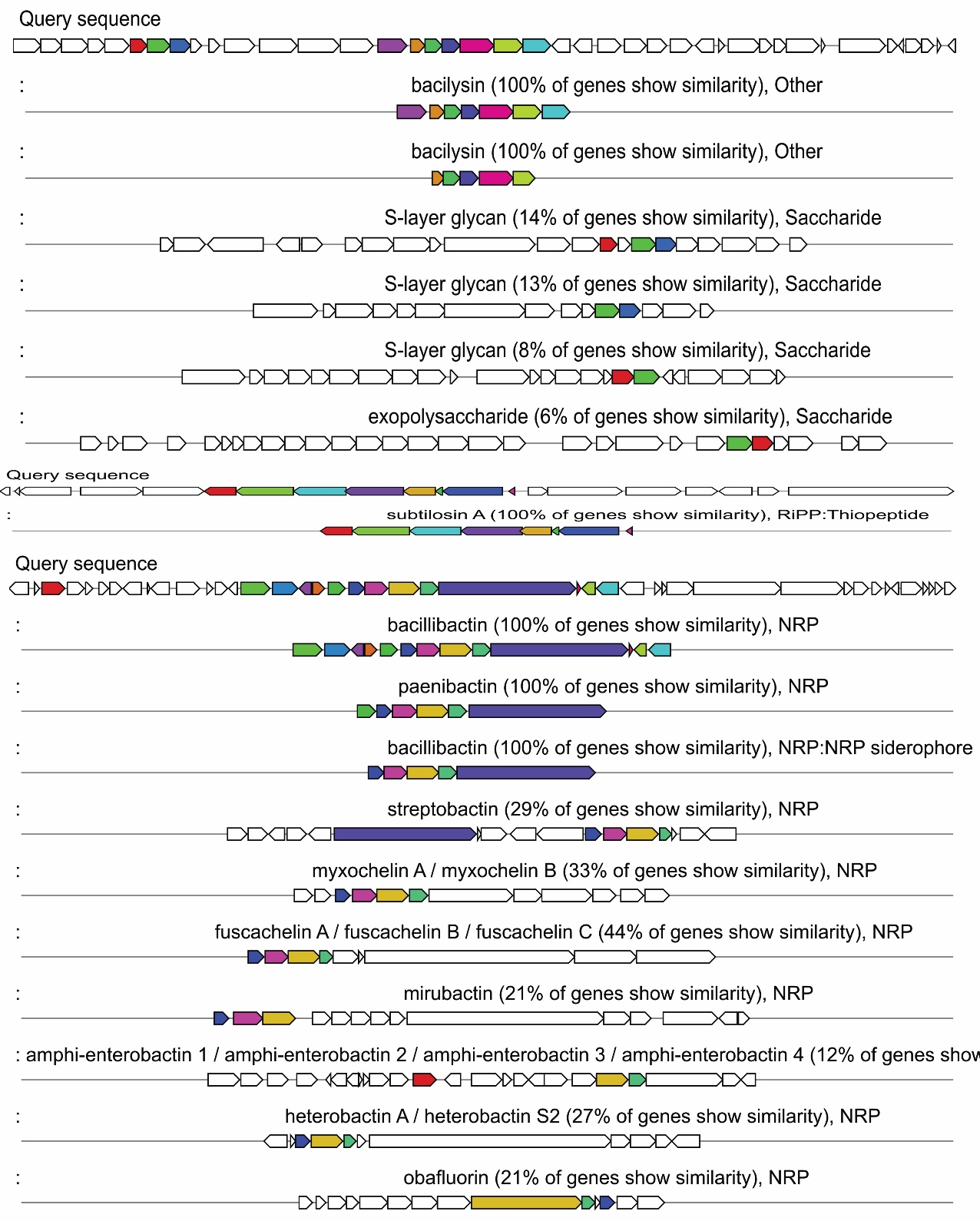


**Fig. S4** Detection of biosynthetic genes


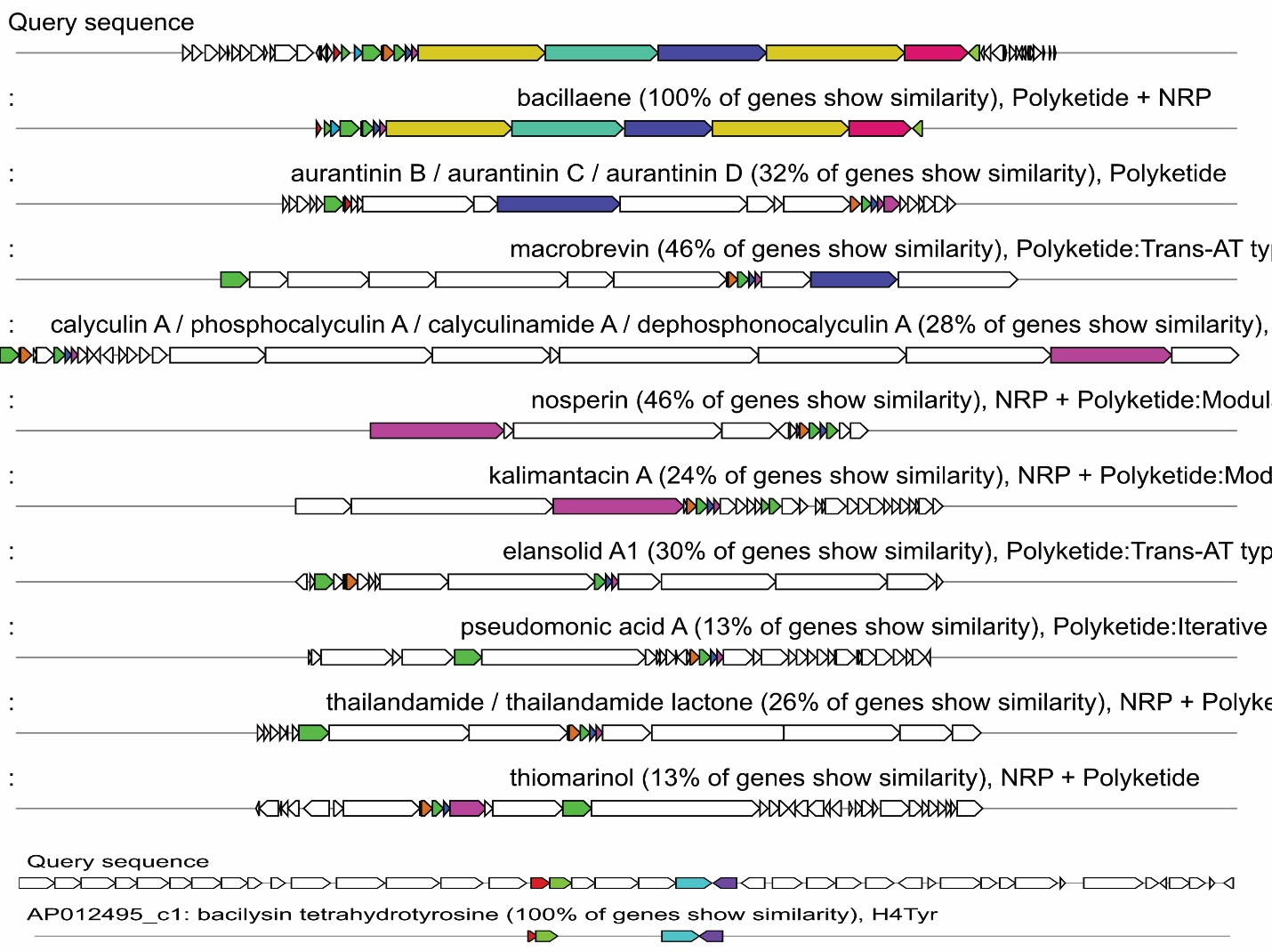


**Fig. S5** Detection of biosynthetic genes


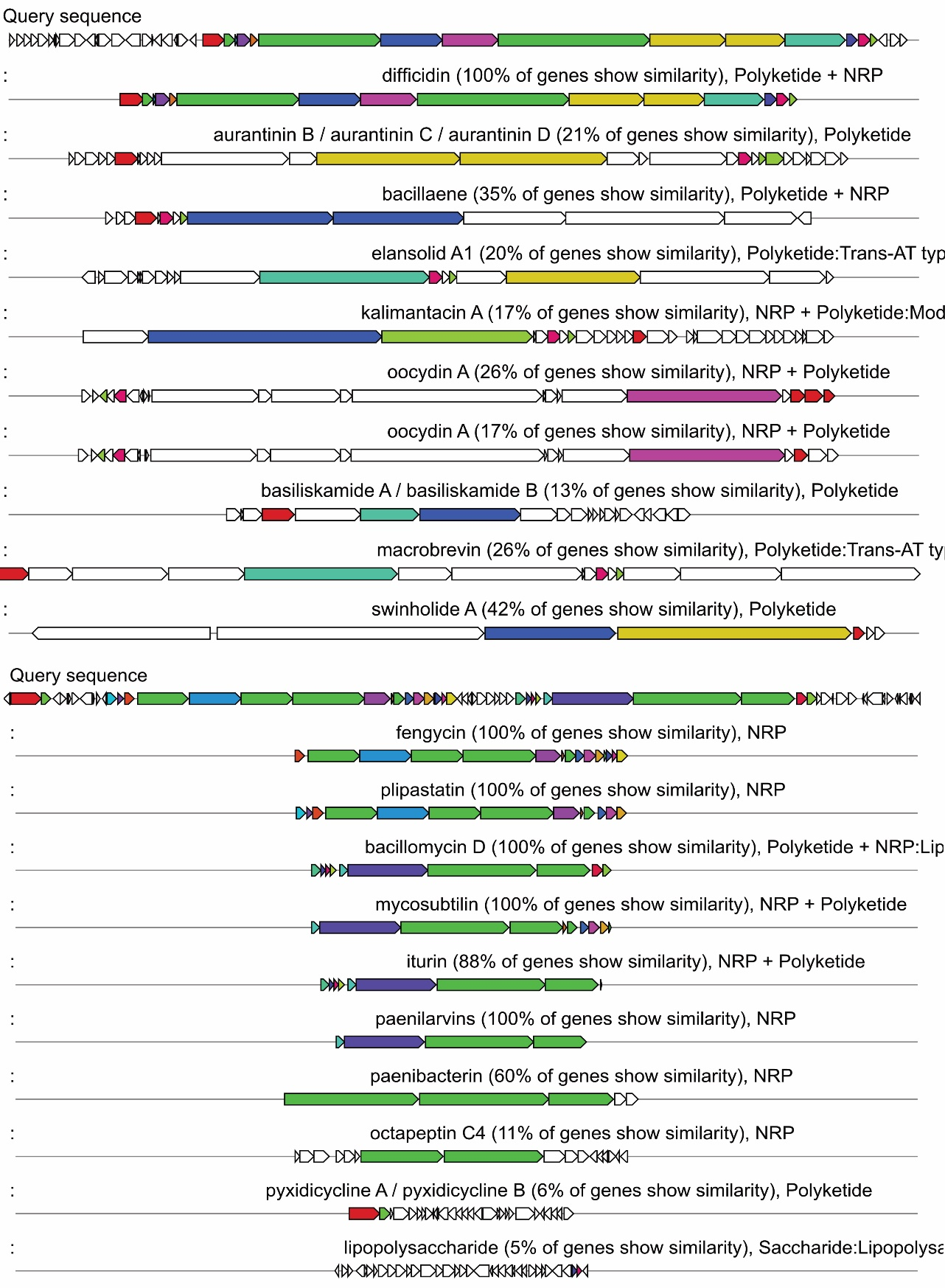


**Fig. S6** Detection of biosynthetic genes


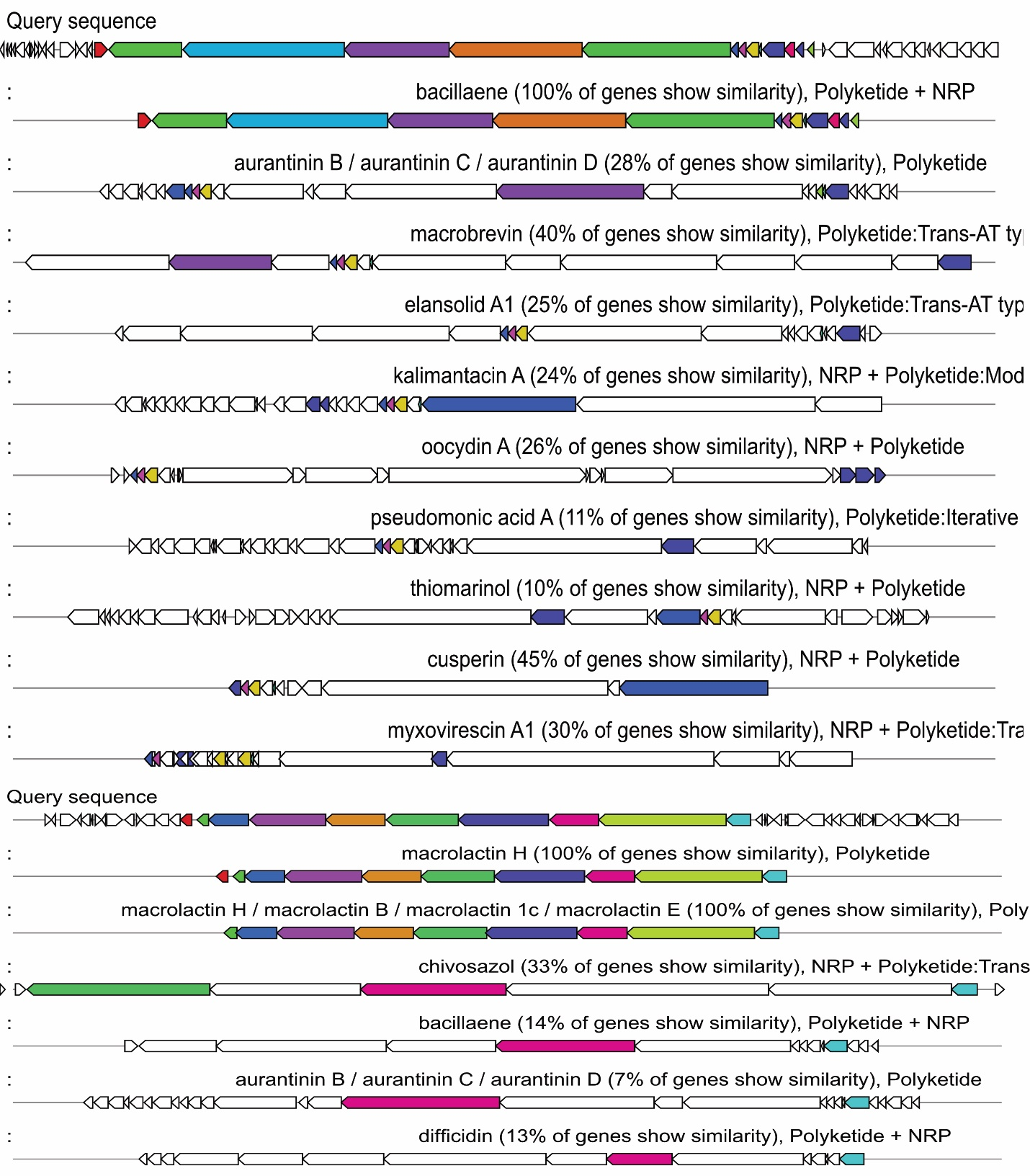


**Fig. S7** Detection of biosynthetic genes


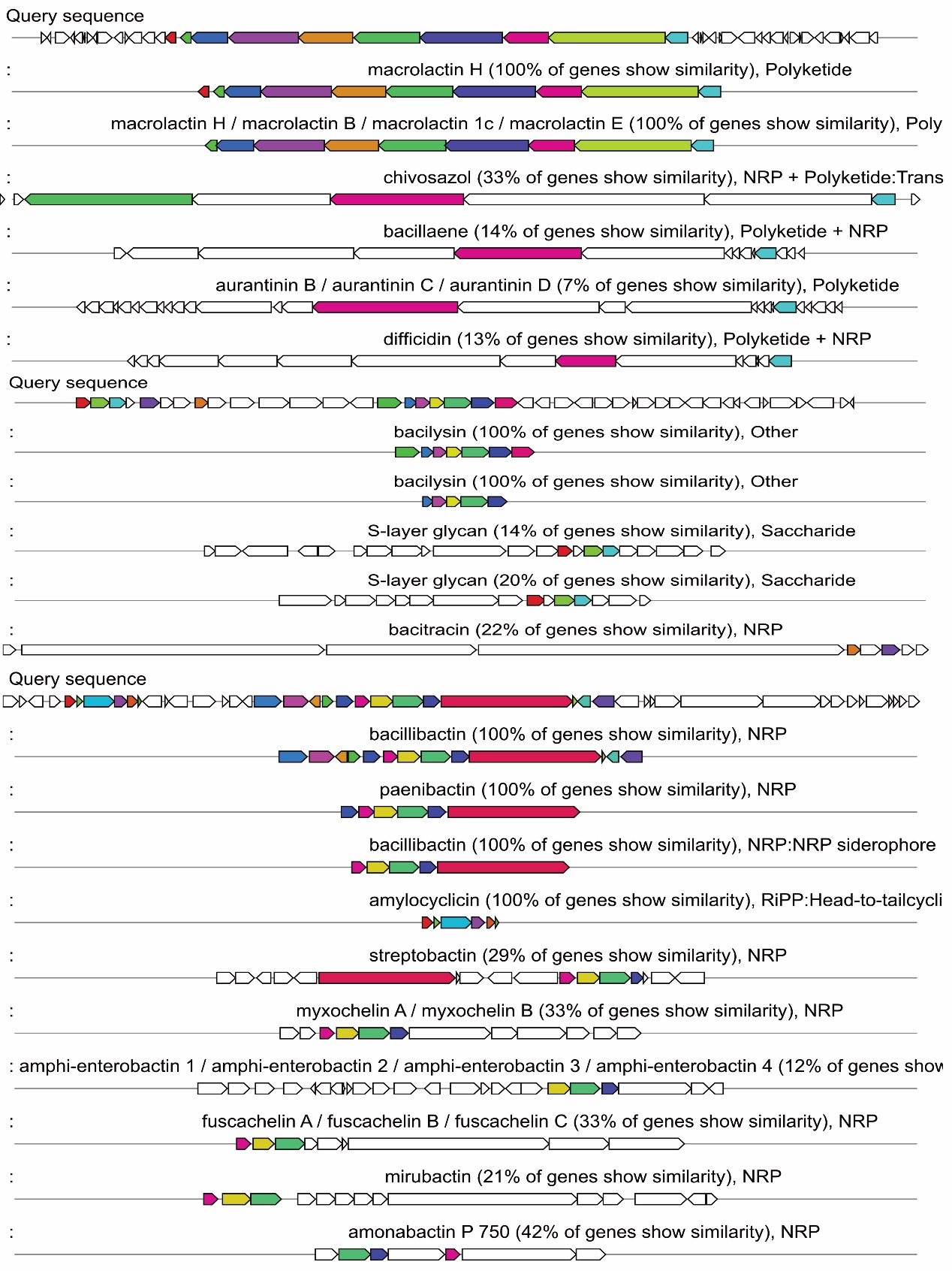


**Fig. S8** Detection of biosynthetic genes


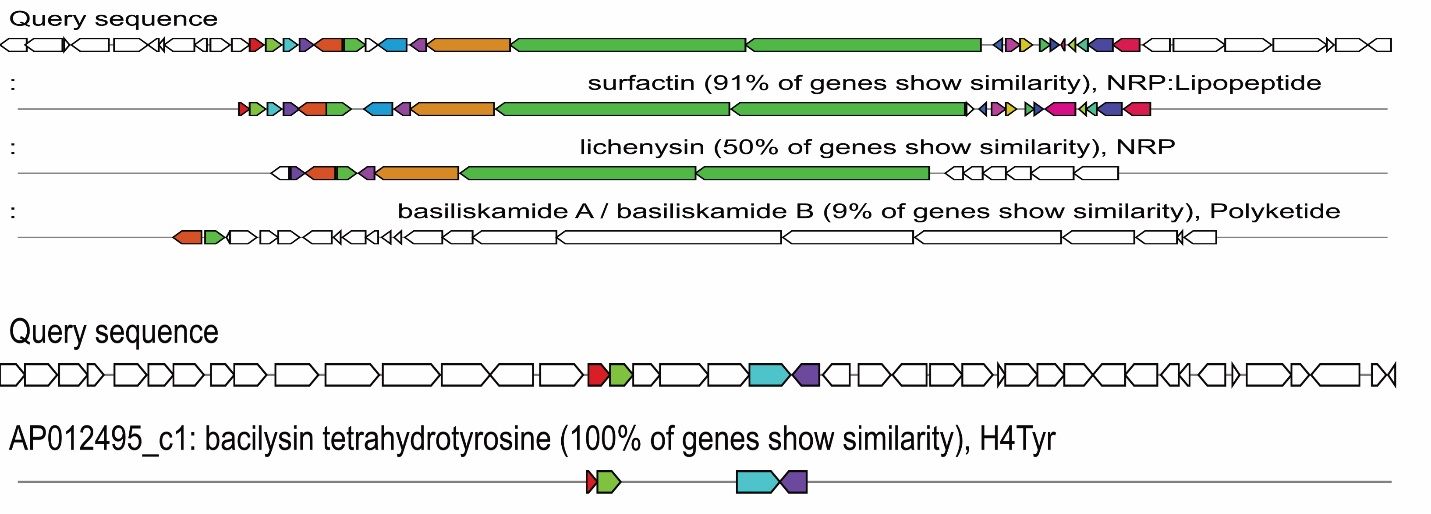


**Fig. S9** Detection of biosynthetic genes


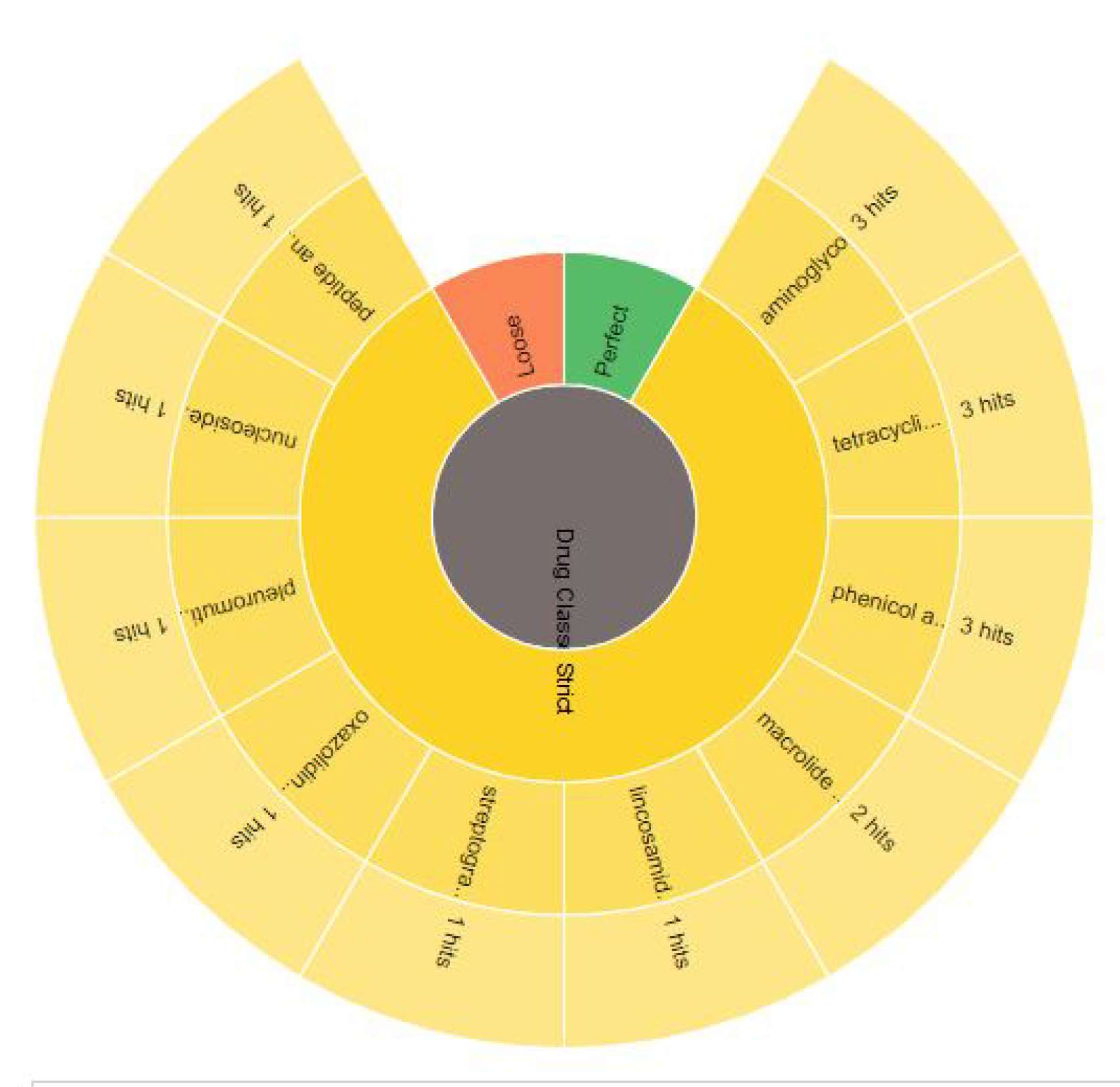


**Fig. S10** Antimicrobial drug class


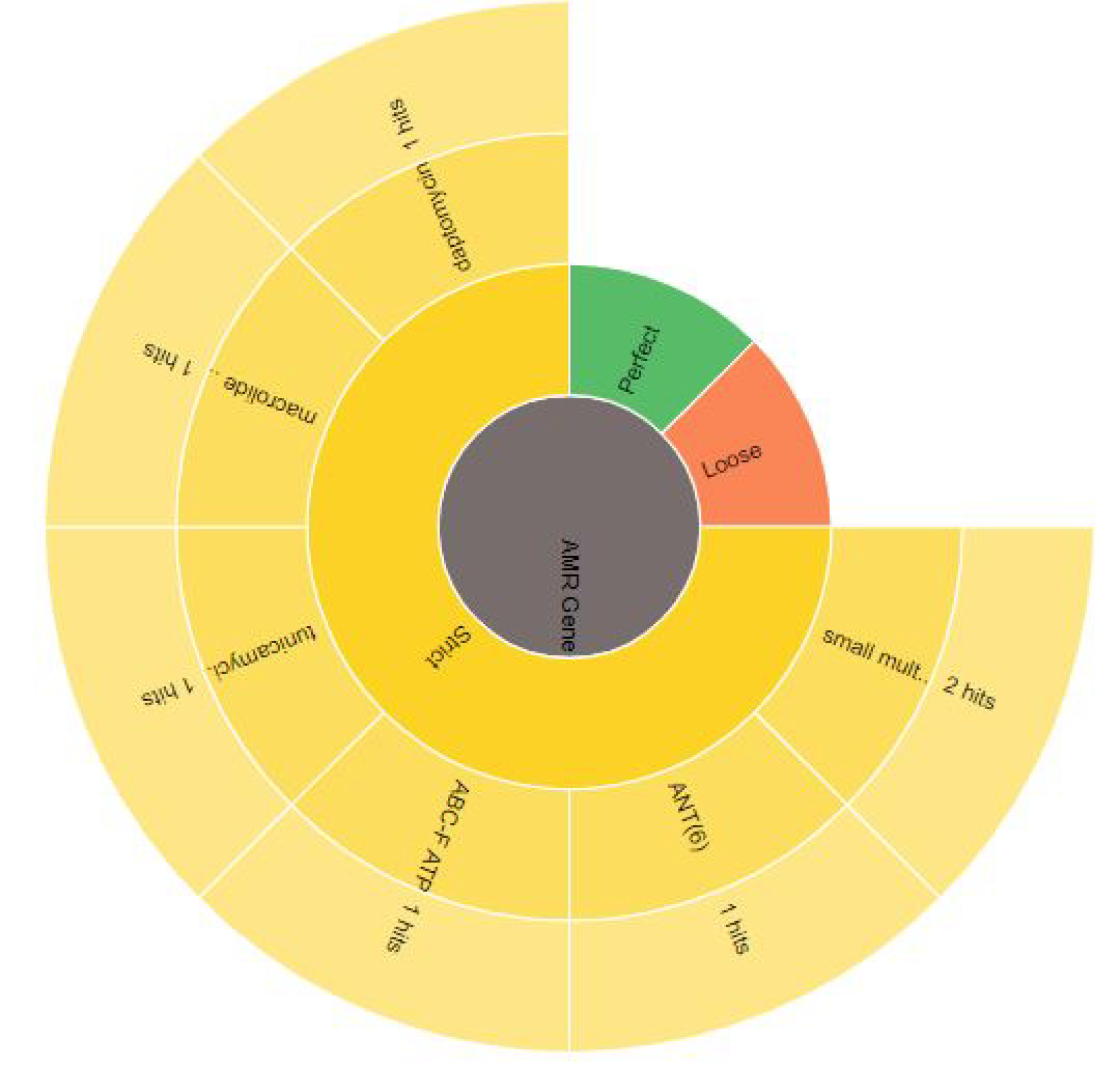


**Fig. S11** Antimicrobial resistance gene family


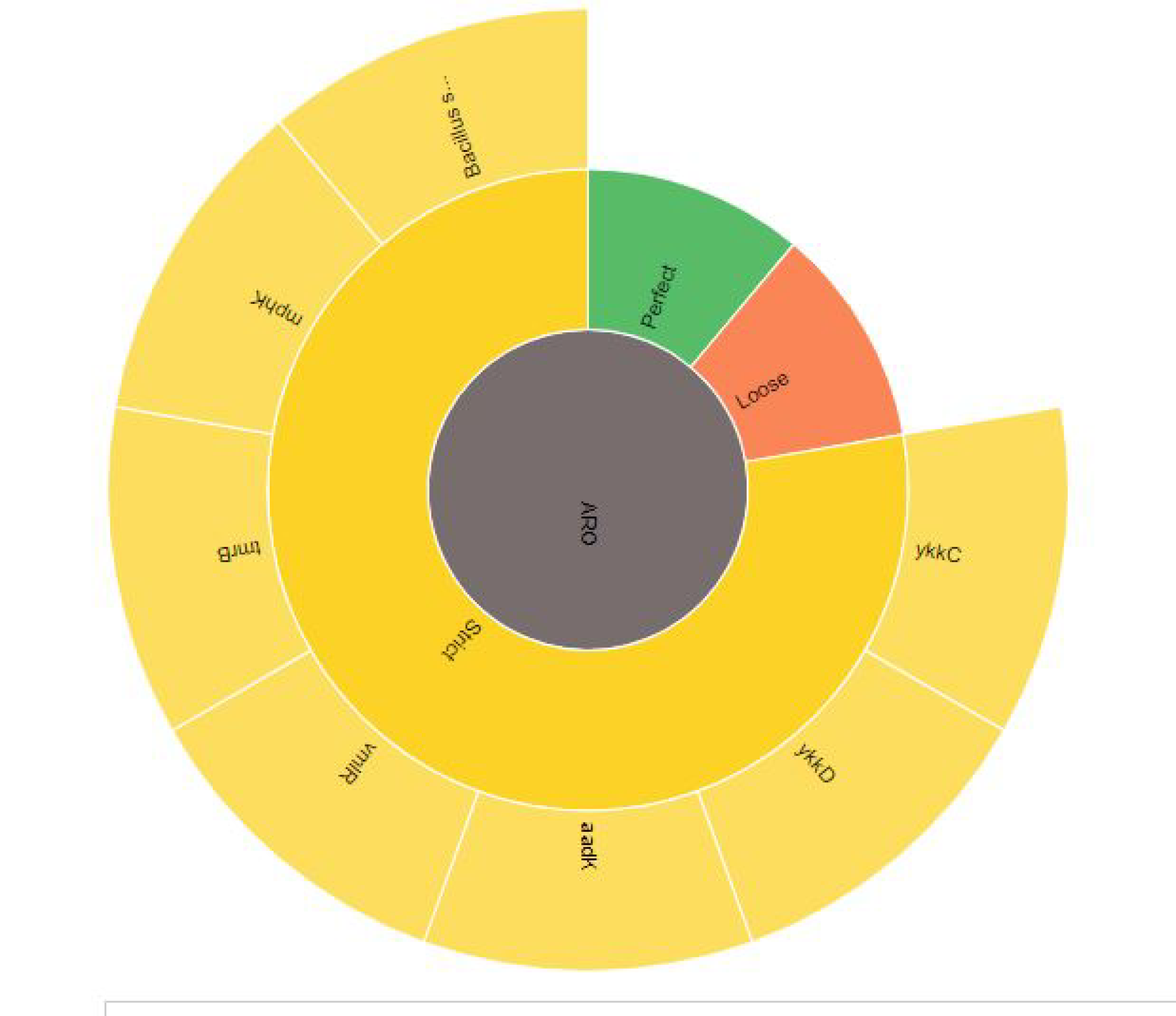


**Fig. S12** Antimicrobial resistance gene


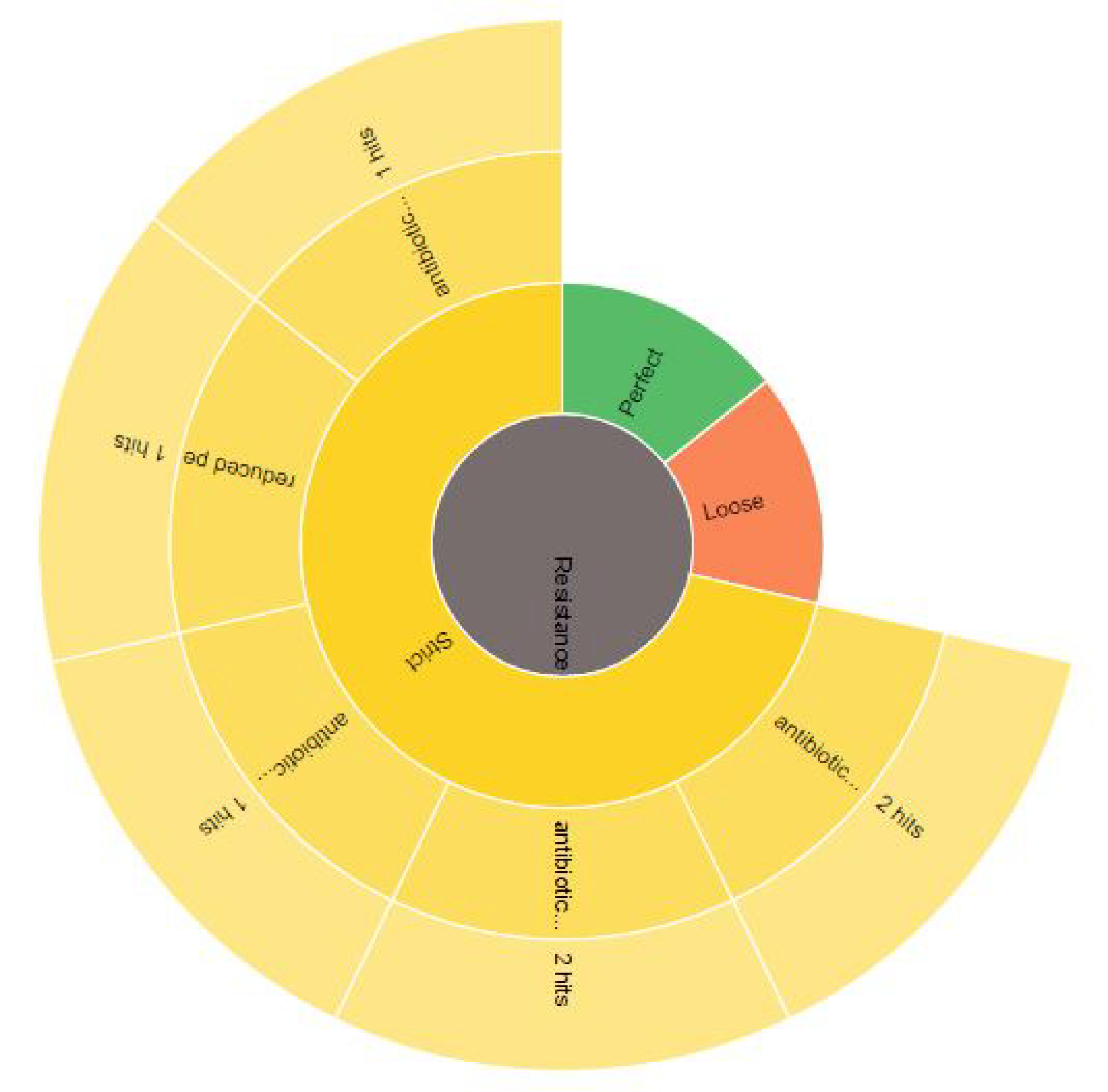


**Fig. S13** Resistance mechanisms


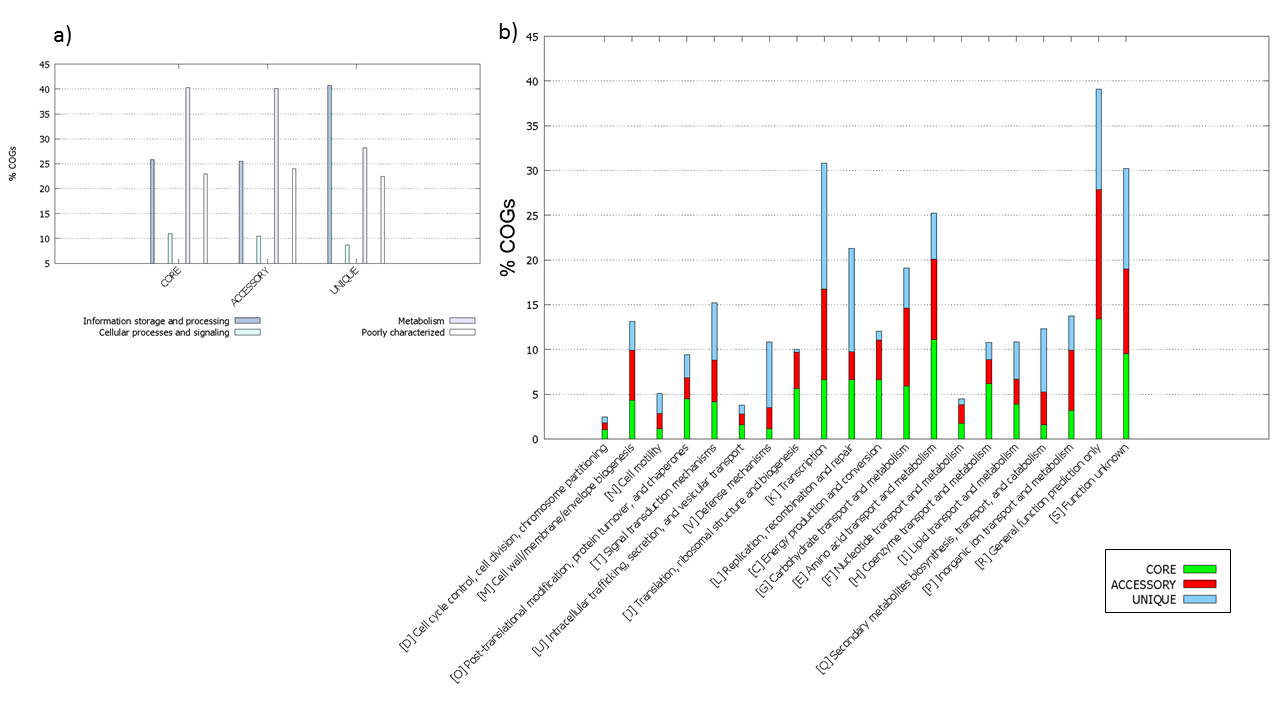


**Fig. S14** COG analysis showing core, accessory, and unique genes


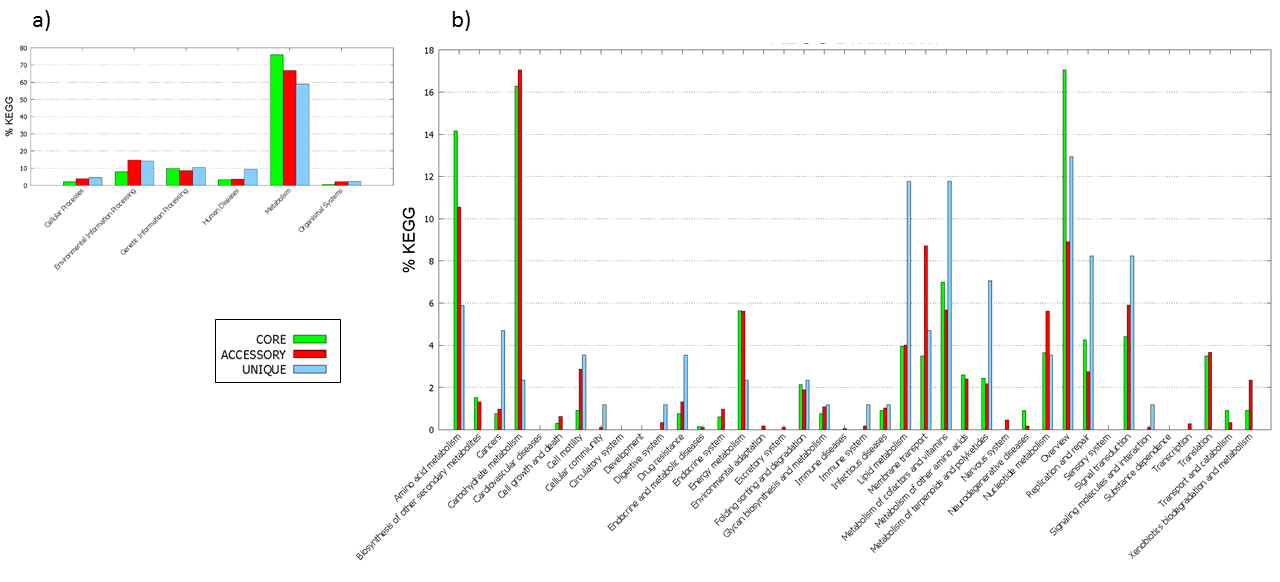


**Fig. S15** KEGG analysis showing core, accessory, and unique genes
